# Muscle transcriptome provides the first insight into the dynamics of gene expression with progression of age in sheep

**DOI:** 10.1038/s41598-021-01848-5

**Published:** 2021-11-16

**Authors:** Reena Arora, Naveen Kumar Siddaraju, S. S. Manjunatha, S. Sudarshan, Mohamed Nadeem Fairoze, Ashish Kumar, Pooja Chhabra, Mandeep Kaur, R. M. Sreesujatha, Sonika Ahlawat, Ramesh Kumar Vijh

**Affiliations:** 1 Animal Biotechnology Division, ICAR-National Bureau of Animal Genetic Resources, G T Road By-Pass, P O Box 129, Karnal, 132001 Haryana India; 2 Karnataka Veterinary Animal and Fisheries Sciences University, Bangalore, 560024 India

**Keywords:** Molecular biology, Transcriptomics, Animal biotechnology, Functional genomics

## Abstract

The dynamic synergy of genes and pathways in muscles in relation to age affects the muscle characteristics. Investigating the temporal changes in gene expression will help illustrate the molecular mechanisms underlying muscle development. Here we report the gene expression changes in skeletal muscles through successive age groups in Bandur, a meat type sheep of India. RNA sequencing data was generated from the *longissimus thoracis* muscles from four age groups, ranging from lamb to adult. Analysis of 20 highest expressed genes common across the groups revealed muscle protein, phosphorylation, acetylation, metal binding and transport as significant functions. Maximum differentiation was observed after 2.5–3 years on transition from lambs to adult. Transcriptional regulation by the TFAP2 transcription factors, IL-6 signaling and PI3K/AKT signaling pathways were enriched in younger animals. The gene-protein network demarcated key interactive genes involved in muscle development and proliferation that can be used as candidates for future research on improvement of muscle characteristics.

## Introduction

Livestock sector plays an important role in the rural economy of India. Sheep and goats contribute substantially to the livelihood of the small and marginal farmers. Small ruminants provide 21.89% of the total meat produced, of which 8.36% is from sheep^1^. Meat quality is an amalgamation of the effects of nutrition, environment and genetic capability. Factors like growth and development of skeletal muscle directly affect the quality and quantity of meat. The palatability of meat is influenced by tenderness and fat content, which are in turn affected by nutrition, muscle characteristics, post mortem events, genetics and the age of the animal^2^. Several studies have evaluated the impact of age on the tenderness and fatty acid profile of muscles in various species^3,4^. The dynamic synergy of genes and pathways in muscles in relation to age of the animal has also been explored in several species^5–7^. In sheep however, there is dearth of related information on lambs and mature animals.

Deep sequencing techniques have significantly broadened our knowledge of global gene expression patterns and regulatory mechanisms in several tissues. Transcriptomic studies have identified regulatory factors involved in muscle growth and meat quality in livestock species^8,9^. The genetic mechanisms underlying growth and development of skeletal muscles of sheep have been investigated using next generation sequencing techniques^5^. Differentially expressed genes for contrasting characteristics have been detected in small as well as large ruminants^8,10^. Such efforts have led to identification of biomarkers for meat quality^11^.

Consumer preferences for Bandur sheep in India led to the investigation that established tenderness and higher backfat content in muscles of Bandur sheep as compared to local sheep found in the same area^12^. Our previous studies have attempted to understand the regulatory molecular mechanisms underlying the muscle traits in Bandur sheep breed in comparison to the local sheep^12,13^. These studies have identified the genes and pathways that may be associated with the muscling traits. Previous studies on goat^6^ and pigs^14^ have provided some insight on the temporal pattern of expression of genes through developmental stages. However, lack of information on the effect of age on the expression of genes in skeletal muscles of sheep has prompted this investigation. The aim of the present study was to compare the gene expression pattern in skeletal muscles through successive age groups in Bandur sheep, a meat type breed of India. The temporal changes in gene expression will help illustrate the molecular mechanisms underlying muscle development in sheep.

## Results

**Figure 1:**
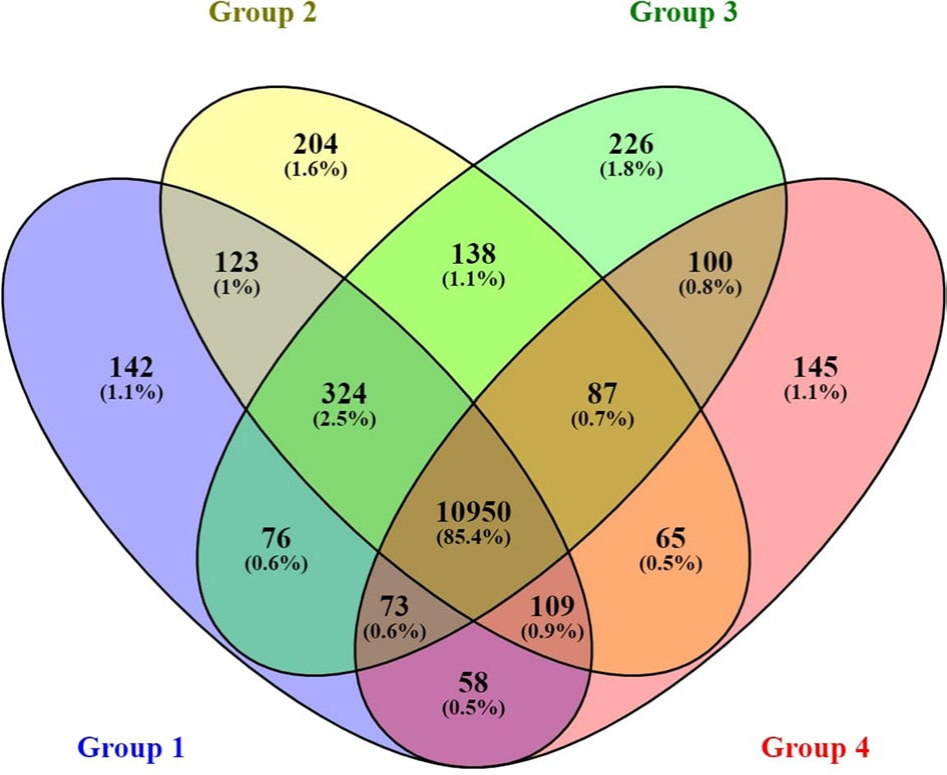
Distribution of transcripts across four age groups in Bandur sheep. Group1 = 2 tooth stage or 1 years; Group 2 = 4 tooth stage or 1.5–2yrs; Group 3 = 6 tooth stage or 2.5–3 years; Group 4 = 8 tooth stage or 3.5–4 years.

Quality control and filtering of raw data resulted in an average of 93,434,064 reads for each library. The processed reads were aligned to the *Ovis aries* reference genome Oar_v4.0 (SAMN00116405). An overall mapping of 94.6% was observed across samples of all age groups (Table S1) while 79–90% of the reads were uniquely mapped. Comparison of the transcript profile revealed 85.4% transcripts common to all groups. The unique transcripts in each group accounted for only 1–1.8% (Fig. 1). The total number of known genes discovered with a minimum threshold of RPKM > 0.01, was 11045, 11168, 10907 and 10514 in group 1, 2, 3 and 4, respectively (Table S2).

### Abundantly expressed genes and their functions

**Figure 2:**
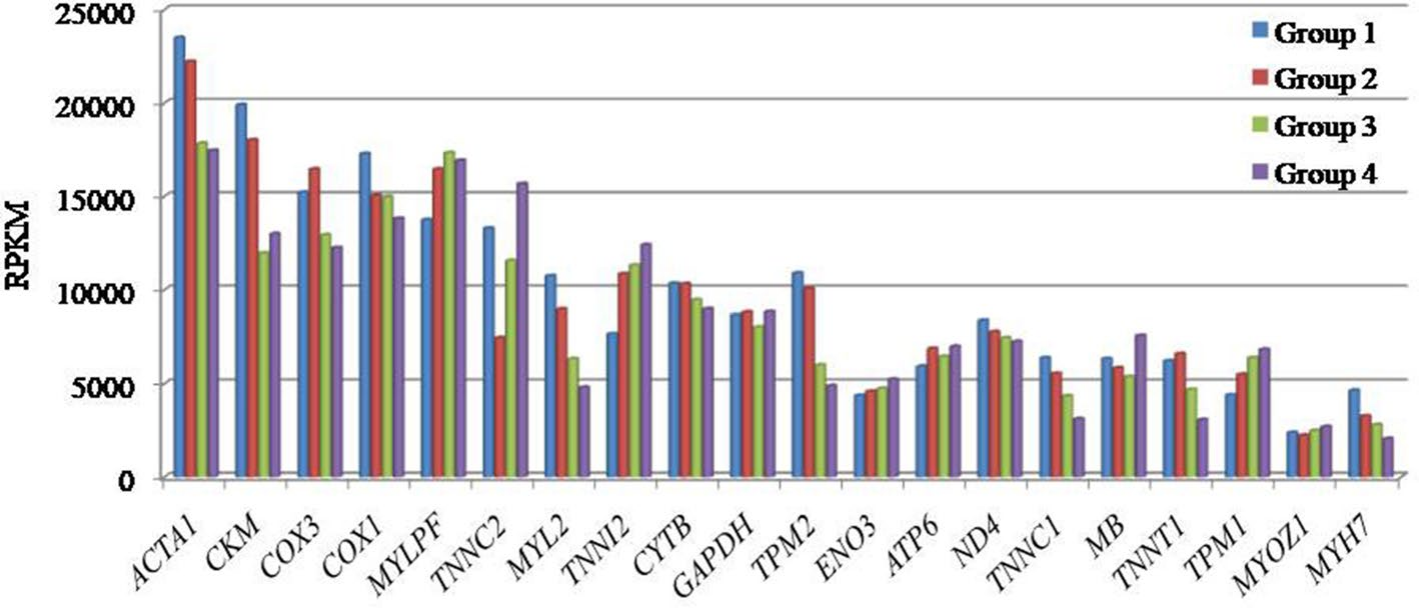
Highest expressed genes with Reads Per Kilobase Million (RPKM) > 2000 across all age groups in Bandur sheep. Group1 = 2 tooth stage or 1 year; Group 2 = 4 tooth stage or 1.5–2 years; Group 3 = 6 tooth stage or 2.5–3 years; Group 4 = 8 tooth stage or 3.5–4 years.

**Figure 3:**
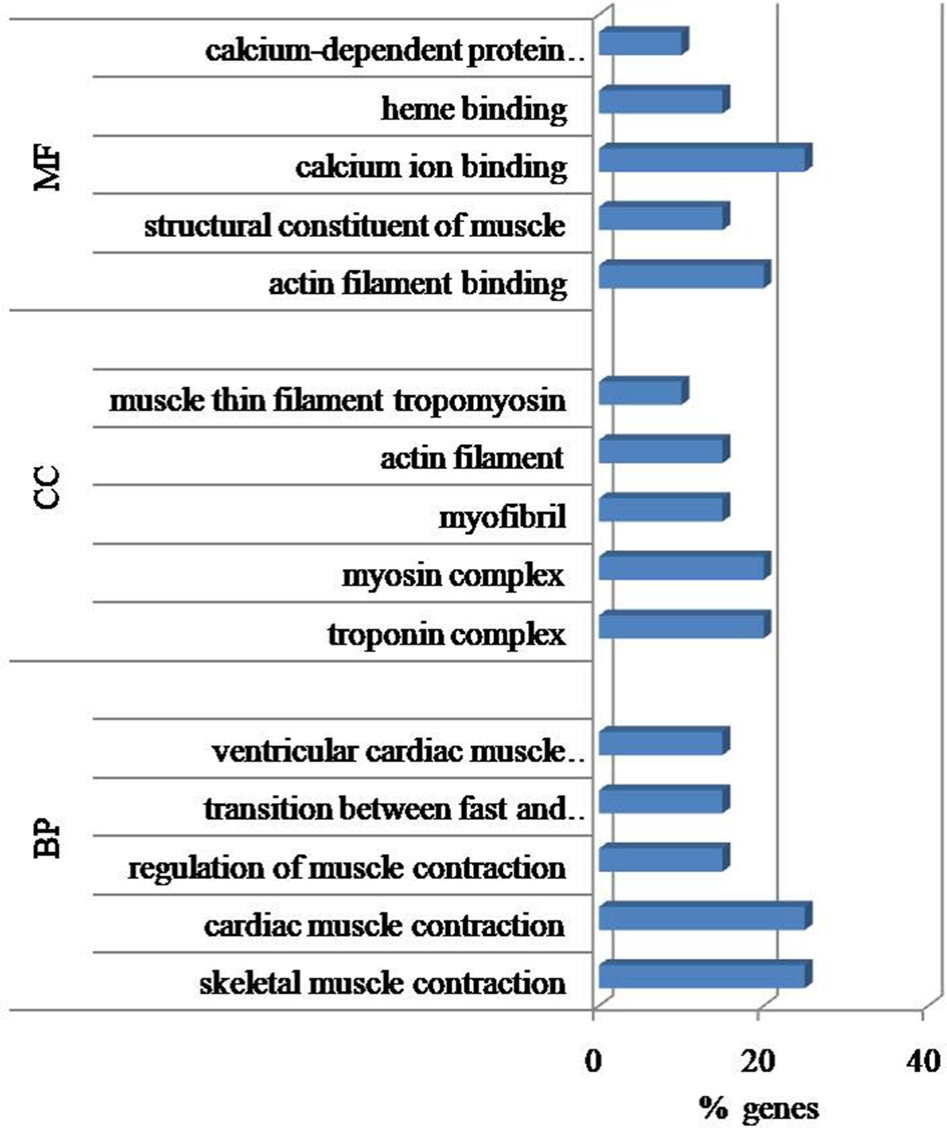
Gene ontologies for 20 highest expressed common genes in all four groups (BP-biological process; CC-cellular component; MF-molecular function). Group1 = 2 tooth stage or 1 yr; Group 2 = 4 tooth stage or 1.5–2 years; Group 3 = 6 tooth stage or 2.5–3 years; Group 4 = 8 tooth stage or 3.5–4 years.

Twenty genes common across all four groups, with expression > 2000 RPKM are shown in Fig. 2. Analysis of the 20 highest expressed genes common across the groups revealed muscle protein, phosphorylation, acetylation, metal binding and transport as significant (p ≤ 0.05) functions. The major biological processes associated with these genes included skeletal muscle contraction, regulation of muscle contraction, muscle tissue morphogenesis, transition between fast and slow fiber, etc. Significant cellular component were troponin and myosin complex, myofibril actin filament and muscle thin filament tropomyosin while actin filament binding, structural constituent of muscle, calcium ion binding were the major molecular functions common in all age groups (Fig. 3). Among these genes, the expression of *ACTA1, COX3, TPM2, MYL2, CYTB2, MYH7, ND4* and *TNNCI* decreased with progression of age. On the other hand, expression of *TNNI2, TPM1* and *ENO3* genes increased with age. Enriched pathways associated with these genes were muscle contraction, glucose metabolism, respiratory electron transport and creatine metabolism (Table S3).

The expression profile of some genes previously associated with meat quality in livestock species^11^ like *HSPB1* and *HSPB8* conspicuously increased after 3 years of age. Expression of *DNAJB5* showed a mild increase whereas that of *HSPA6* decreased with age. Transcription of *CAPN3* also exhibited a very slight increase with advancement of age in investigated animals. Other genes like *FABP3, CAPN1* and *CAST* did not express prominent difference across the groups. Similarly, collagen genes are also known to influence meat quality^15^. Although the expression of some collagen genes, namely, *COL15A1, COL23A1, COL4A2, COL6A2, COL1A1, COL4A1, COL1A2* and *COL3A1* was observed to be quite low (≤ 7.3 RPKM), their expression was highest in young animals and decreased considerably with age.

### Differentially expressed genes and enriched pathways

**Figure 4:**
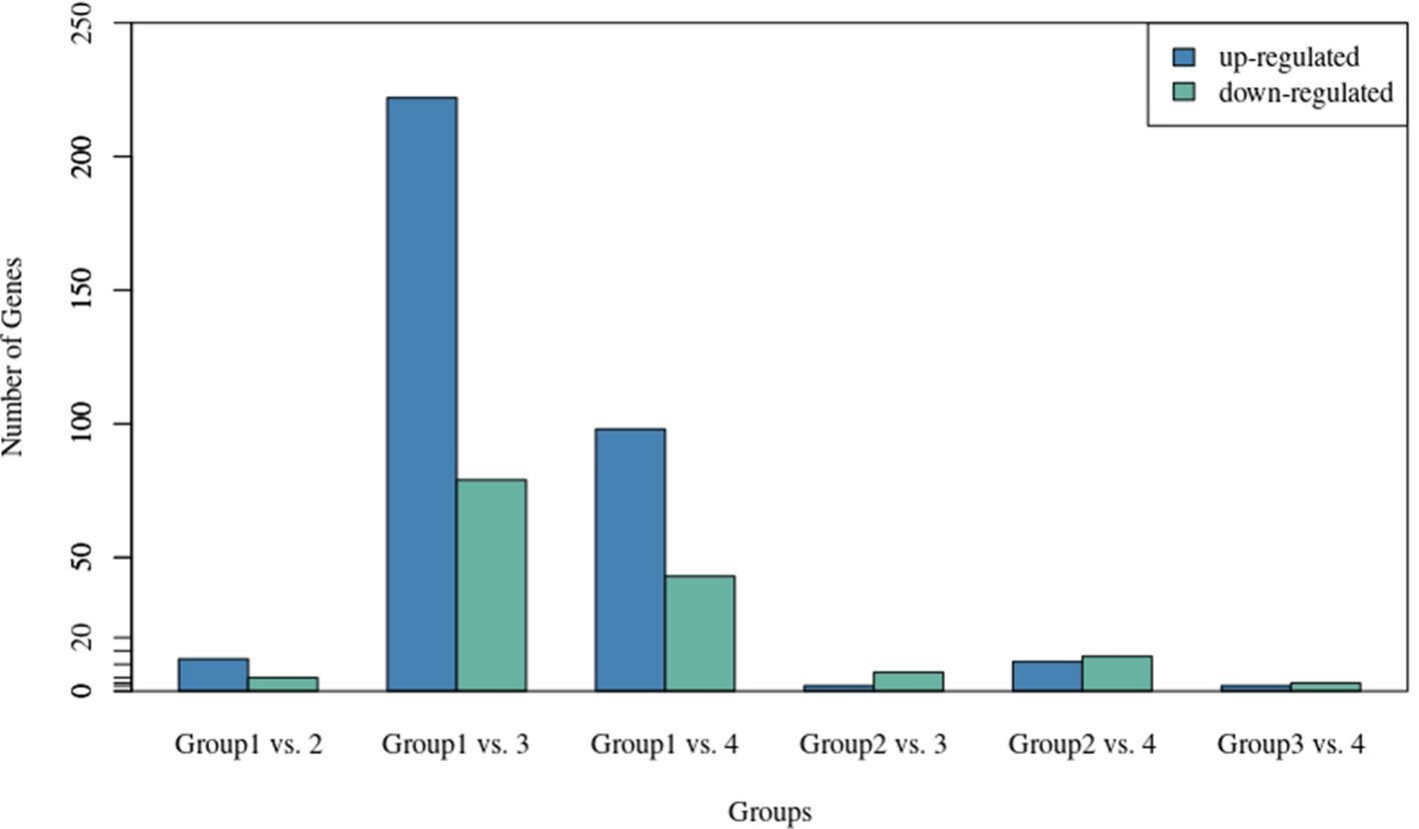
Number of differentially expressed genes between different combinations of age groups. Group1 = 2 tooth stage or 1 years; Group 2 = 4 tooth stage or 1.5–2 years; Group 3 = 6 tooth stage or 2.5–3 years; Group 4 = 8 tooth stage or 3.5–4 years.

**Figure 5:**
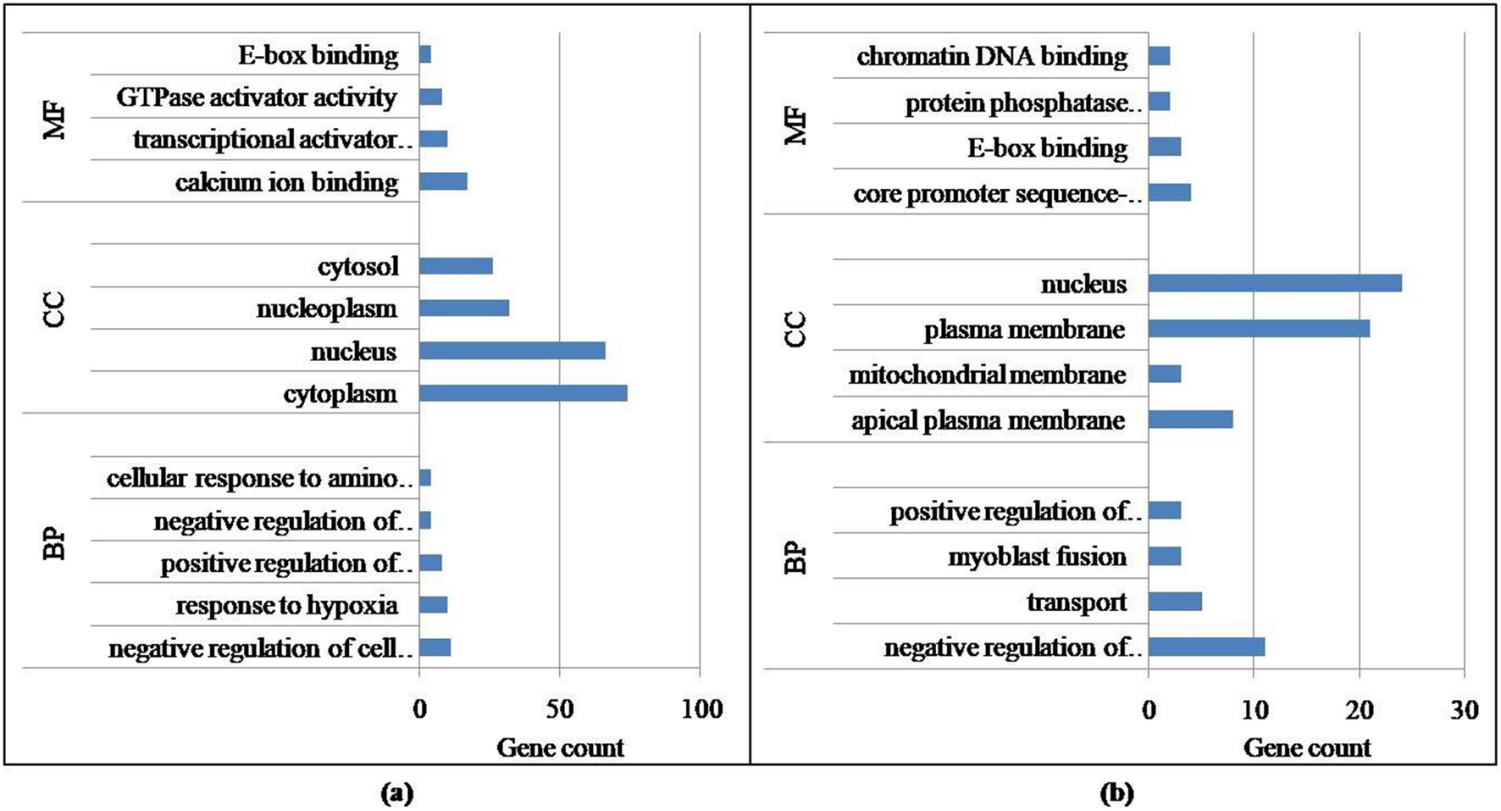
Functional analysis of differentially expressed genes between (**a**) Group 1 and 3 and (**b**) Group 1 and 4. BP-Biological process; CC-Cellular components; MF-Molecular function. Group1 = 2 tooth stage or 1 years; Group 3 = 6 tooth stage or 2.5–3 years; Group 4 = 8 tooth stage or 3.5–4 years.

Differential expression of genes was analyzed across all combinations of the four age groups. Considerably larger number of genes was observed to be differentially expressed between Group1–3 and Group1–4 only, which were therefore used for further analysis (Fig. 4). There were 301 significantly differentially expressed genes in Group 1 versus 3 (p_adj_ < 0.05; FDR < 0.05), followed by 141 in Group 1 versus 4. The up- and down-regulated genes observed in Group 1 versus 3 were 222 and 79, respectively, whereas 98 up-regulated and 43 down-regulated genes were identified in Group 1 versus 4 (Table S4). Gene ontology analysis revealed negative regulation of cell proliferation, response to hypoxia, positive regulation of GTPase activity, negative regulation of cysteine-type endopeptidase activity involved in apoptotic process, calcium ion binding etc., as significant functions (p_adj_ < 0.05) associated with differentially expressed genes in Group 1 versus 3 (Fig. 5a). Significant pathways associated with the up-regulated genes were transcriptional regulation by the TFAP2 (AP-2) family of transcription factors, IL-6 signaling, PI3K/AKT signaling, ECM proteoglycans, muscle contraction etc. (Table S5), while down-regulated genes were related with transcriptional regulation by RUNX1 and RUNX3. Similarly, enriched functions in Group 1 versus 4 included regulation of transcription from RNA polymerase II promoter, myoblast fusion, regulation of epithelial to mesenchymal transition and protein phosphatase regulator activity (Fig. 5b). The top canonical pathways identified again were transcriptional regulation by the TFAP2 (AP-2) family of transcription factors, IL-6 signaling, PI3K/AKT signaling. Other important pathways included MAPK1 and MAPK3 activation, DAG and IP3 signaling and PKA activation (Table S6).

### Validation of RNAseq data by RT-qPCR

**Figure 6:**
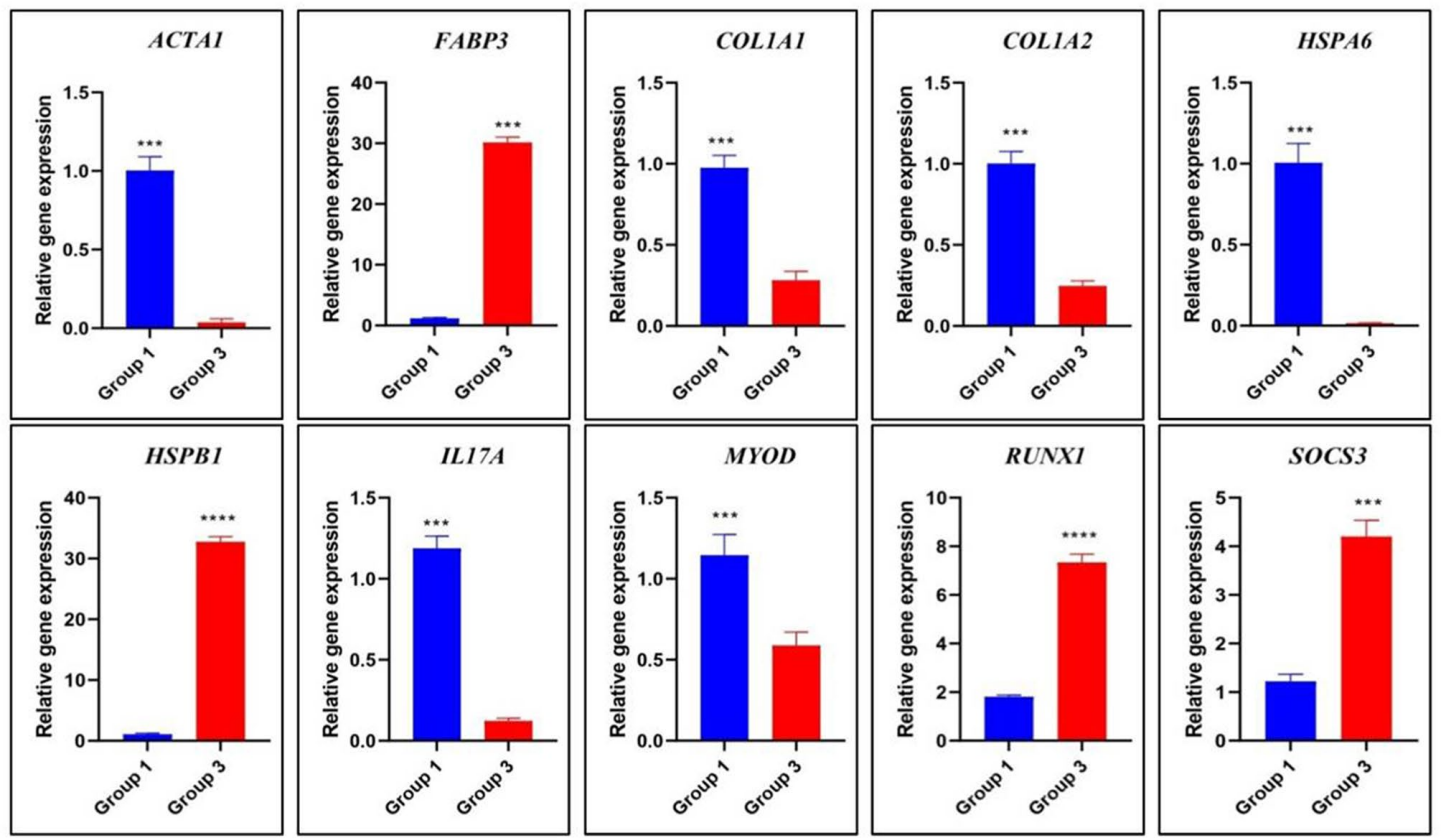
Relative expression of differentially expressed genes between Group 1 and Group 3 by quantitative PCR. Group1 = 2 tooth stage or 1 years; Group 3 = 6 tooth stage or 2.5–3 years.

**Figure 7:**
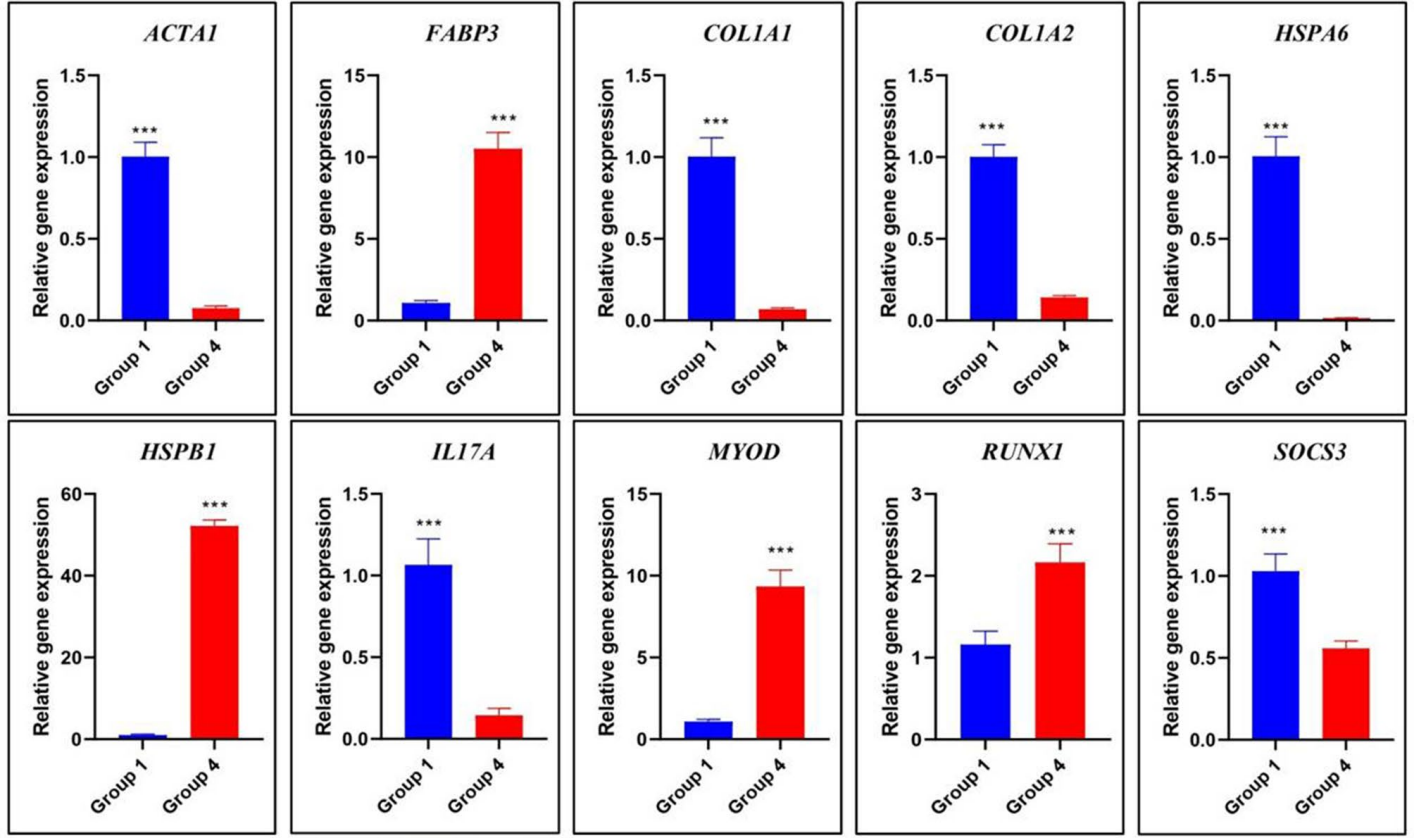
Relative expression of differentially expressed genes between Group 1 and Group 4 by quantitative PCR. Group1 = 2 tooth stage or 1 years; Group 4 = 8 tooth stage or 3.5–4 years.

Differentially expression of genes in Group 1 versus Group 3 and Group 1 versus Group 4 was validated by RT-qPCR. The magnitude of relative expression of the selected genes namely *ACTA1, FABP3, COL1A1, COL1A2, HSPA6, HSPB1, IL17A, MYOD, RUNX1* and *SOCS3* was observed to be similar to that derived from RNAseq data (Figs. 6 and 7).

### Gene—protein interactions

**Figure 8:**
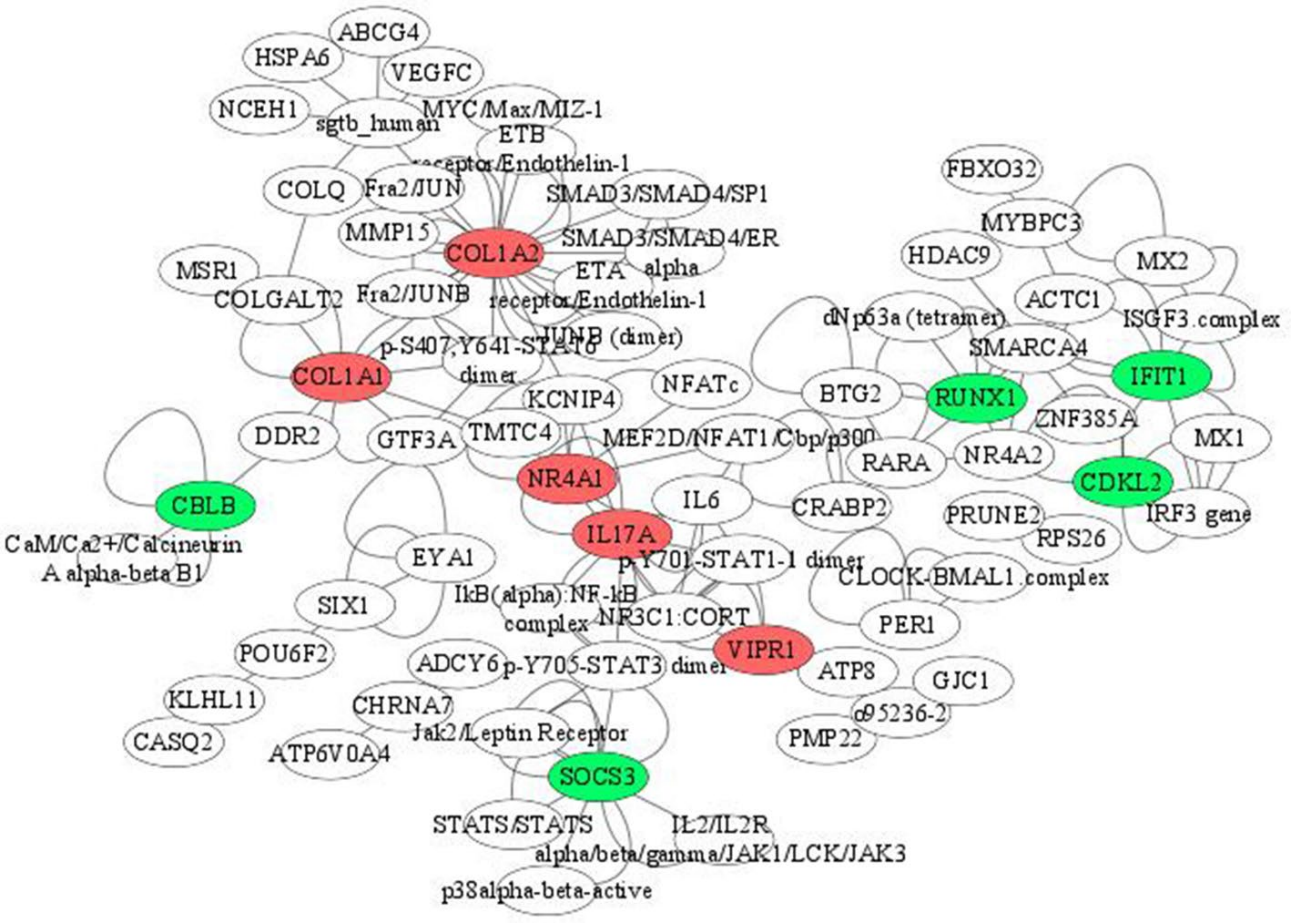
Highly connected differentially expressed genes identified by comparison of age Groups 1 and 3. The top 10 highly connected genes have been highlighted (red: up-regulated; green: down-regulated). Group1 = 2 tooth stage or 1 years; Group 3 = 6 tooth stage or 2.5–3 years.

**Figure 9:**
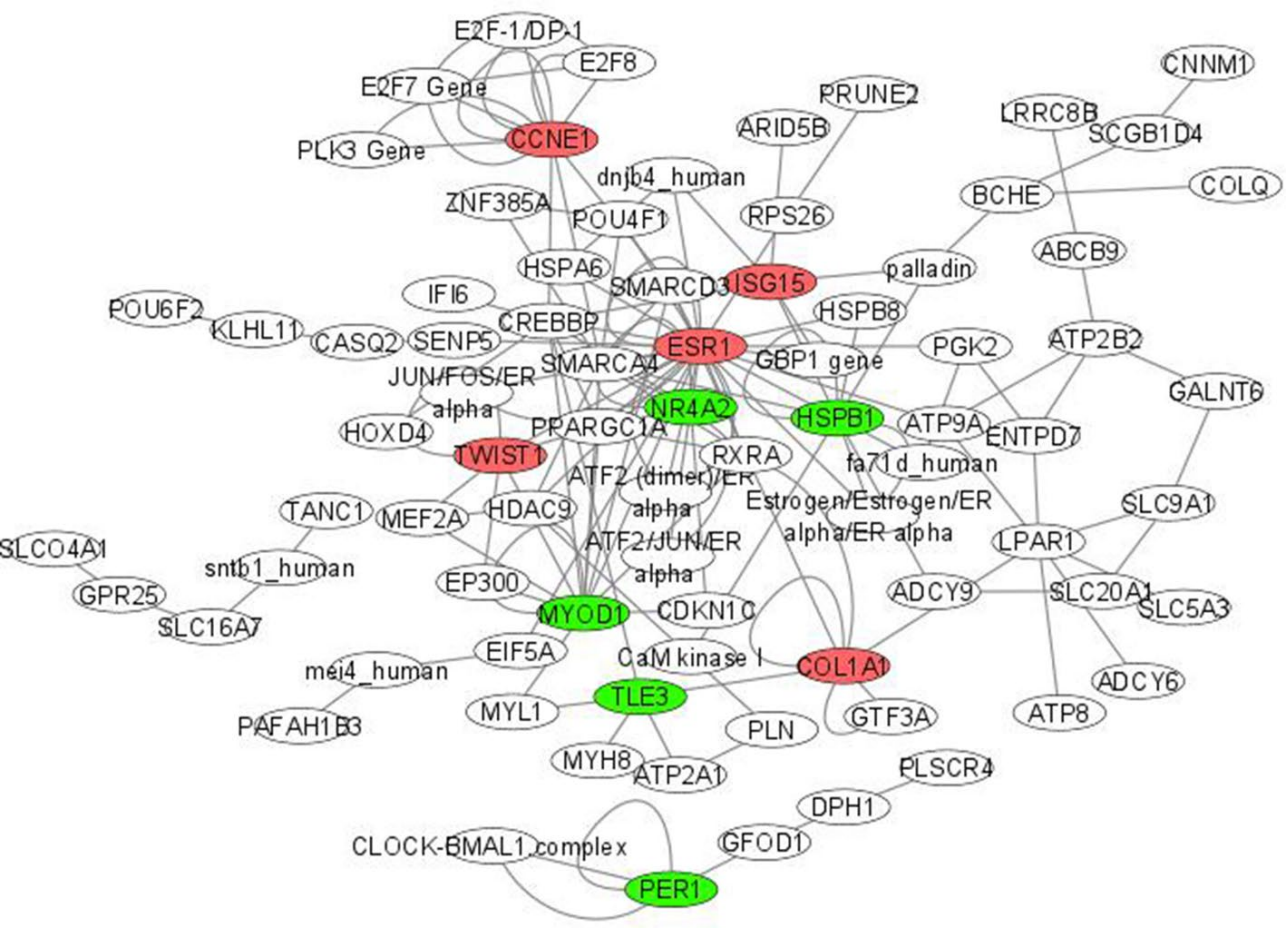
Highly connected differentially expressed genes identified by comparison of age Groups 1 and 4. The top 10 highly connected genes have been highlighted (red: up-regulated; green: down-regulated). Group 1 = 2 tooth stage or 1 years; Group 4 = 8 tooth stage or 3.5–4 years.

To identify highly connected genes from the set of differentially expressed ones, a gene—protein network was constructed for each comparative Group 1 versus 3 and Group 1 versus 4 (Figs. 8 and 9). The central genes with 5 or more interactions identified for Group1 versus 3 included *COL1A2, SOCS3, IL17A, COL1A1, IFIT1, NR4A1, RUNX1, CDKL2, CBLB* and *VIPR1.* Whereas *ESR1, CCNE1, MYOD1, HSPB1, COL1A1, NR4A2, TWIST1, ISG15, PER1* and *TLE3* were the most interactive hub genes identified in the network for Group 1 versus 4. Among these genes *COL1A2, IL17A, COL1A1, NR4A1, VIPR1, ESR1, CCNE1, TWIST1* and *ISG15* were up-regulated, while the remaining were down-regulated. *COL1A1* was common in both the networks. Some other important genes identified in the network were *HSPA6, LPAR1, EIF5A* and *PPARGC1A*.

## Discussion

Muscle traits relevant to meat quality are modulated by age, nutrition, genetics and environmental factors which in turn affect the structure and composition of muscle fibres as well as intramuscular fat. Differential expression of genes in muscles between different breeds in relation to feed, carcass and muscle properties has been examined by several studies^8–10,12^. It is well established that muscle characteristics are affected by age^16^. However, investigating the simultaneous change in gene expression with age in skeletal muscles of sheep has not garnered much interest. The present study therefore, explored the pattern of gene expression with progression of age in skeletal muscles of Bandur sheep. The sarcomere proteins, mainly alpha-actinins, myosin heavy chains and Z disc form the basic units of muscle fibres and are coded by *ACTA1, ACTN2, ACTN3, MYH4, MYH7 and MYOZ* genes^17,18^*.* The troponin complex modulates the interaction between actin and myosin during muscle contraction^19^. *TNNI2* and *TNNC2* encode for their respective troponin isoforms and are known to play a key role in muscle composition and aging^20^. The genes mainly involved in muscle contraction and muscle fibre composition (*ACTA1, ACTN2, ACTN3, ANKRD1, TCAP, NRAP, MYH4, MYH8, TNNI2, TNNC2, MYOZ1, LDB3, TPM1*) were abundantly expressed irrespective of age group in our study. Earlier investigations have revealed no significant effect of sex on muscle fibre type, however, the fibre diameter was affected^21^. Hence, further research on gene expression in *longissimus thoracis* muscles, across both rams and ewes is warranted. Besides structural components of muscles the highly expressed genes also represented glycogen metabolism and storage (*LDHA, PYGM, PGAM2*) as well as energy metabolism (*COX1, COX3, CKM, ND4*). The calpains are another family of genes associated with muscle tenderization^22^. The calpain-calpastatin activity was reported to increase with age in beef cattle^23^. It is well established that the activity of calpains is enhanced during aging^24^. A slight increase in the expression of *CAPN3* with age was also detected in Bandur sheep. The domination of expression of these genes in our study reinforces their fundamental role in muscle physiology.

Major pathways identified by differential analyses also had relevance to myogenesis. During muscle development, the transition from epithelial to mesenchymal cells is known to be regulated by TFAP2^25^. Studies on mice have demonstrated that cytokines of the IL-6 family play a crucial role in regulating myogenesis^26^. It is interesting to note that this activity is in turn dependent on MAPK and NFκβ signaling^27^. Recent studies on Bandur sheep have also reported the enrichment of the PI3K-Akt and MAPK pathways that are associated with hypertrophy as well muscle differentiation^12,13^. These pathways were enriched in the young animals.

As expected, lesser differentiation of gene expression was observed between successive age groups. Differential expression was prominent after 2.5–3 years in Bandur sheep reflecting transition from lamb to adult sheep. Major differences observed between lambs and adult sheep muscles were evident in the expression of genes associated with muscle structure, growth and lipid metabolism. The gene-protein network demarcated important interactive hub genes that may be involved in age related muscle development in sheep. The hub genes enriched in younger animals included *COL1A2, COL1A1, TWIST1, NR4A1, VIPR1, CCNE1, ESR1, IL17A* and *ISG15.*It is worth mentioning that these highly connected genes identified in our study are known to be involved in muscle growth and regeneration. Collagens form the major connective tissue that imparts structural strength to muscles^28^. The crosslinking between the collagen molecules increases with age resulting in increased toughness of meat in adult animals^18^. The expression of *COL1A1* and *COL1A2* was observed to be decreased in bovine fetuses that were myostatin deficient^29^. A decrease of collagen expression in older animals was also observed in mice^30^. Several studies have associated the significance of expression of collagen type 1 gene in muscle composition and regulation of intra muscular fat in pork^31,32^. Age affects the structure and distribution of collagen in the extracellular matrix of skeletal muscle^15^, which is also reflected in the change in expression of collagen precursor genes in skeletal muscles of Bandur sheep. *TWIST1* is another gene encoding a transcription factor that is involved in the early growth of skeletal muscles but its function in adults is less explored^33^. It is also known to inhibit DNA binding of *MYOD1* during myotube formation^34^. Other genes with higher expression in lambs that are associated with muscle growth include *NR4A1* and *VIPR1.* Experimental evidence using NR4A1 knockout mice suggests its association with growth and proliferation of muscle cells^35^. Studies on mouse myoblasts revealed NR4A1 as an important factor regulating myoblast differentiation^36^. *VIPR1* is a G-protein coupled receptor expressed in the skeletal muscles that may be involved in muscle atrophy/hypertrophy^37^. Further, experimental evidence supports the role of the estrogen receptor (*ESR1*) in muscle strength by modulation of myosin regulatory light chain in mice^38^. The *ESR1* gene regulates expression via the estrogen signaling pathway. The *IL17A* gene, on the other hand has been reported to activate PPARγ which is involved in increased adipogenesis^39^. In addition, NR4A receptor family in conjunction with *CRTC2* and *PPARγ* assists in myogenesis and adipogenesis via the CREB pathway^40^. Thus, most of the highly connected genes identified in Bandur lambs were associated with growth, proliferation and adipogenesis.

Notable hub genes in older or mature animals included *MYOD1, RUNX1, TLE3. SOCS3, CBLB, HSPB1, IFIT1, CDLK2, NR4A2* and *PER1.* Myoblast differentiation is orchestrated by an assortment of muscle-specific regulatory factors. *MYOD1*, a myogenic regulatory factor and an important marker for myoblast development, facilitates myogenesis during embryogenesis. MYOD1 along with RUNX1 and TFAP1 transcription factors, has been associated with myoblast regeneration or proliferation^41^. Although the function of MYOD1 in adult muscle is less investigated, there are some reports that have established its role in growth and proliferation in adult muscles^42,43^. *RUNX1* has also been suggested to contribute to myotube generation in humans^44^ and its expression is activated by muscle damage^41^. TLE is a family of transcriptional co-factors that are involved in cell differentiation. The transcriptional activity of MyoD has also been reported to be regulated by *TLE3*^45^. Another important gene *SOCS3*, identified in this study has been linked to myogenic differentiation^46^. Previous reports suggest the role of *CBLB* gene in skeletal muscle atrophy in mice by negative regulation of growth factors during cell differentiation and development^47^.

The small heat shock proteins (Hsps) long associated with heat stress, are being increasingly implicated in cellular senescence and apoptosis^48^. The *HSPB1* is highly expressed in muscle tissues and codes for Hsps^49^. The Hsps are anti-apoptotic and prevent the muscles from denaturation, which has implications in meat tenderness^50^. Several studies have reported the involvement of *HSPB1* in meat tenderness in cattle^51,52^. Age related expression of genes associated with meat quality detected considerable increase in the expression of *HSPB1* and *HSPB8* in older age (3.5–4 years) in Bandur sheep. A similar increase in hspb1 protein was observed in the skeletal muscles of chicken^53^. Although much information on *HSPB8* and aging are not available, it is known to be involved in protein damage through translational arrest and autophagy^54^. Previous studies on sheep have reported no significant effect of sex on the tenderness of *longissimus thoracis* muscles^55^. Therefore, it may be speculated that a similar expression of hsps could be expected across both sexes.

The role of some of the identified hub genes is not very well defined in skeletal muscles. These include the interferon stimulated genes *ISG15* and *IFIT1* that are suspected to be involved in muscular diseases^56^. Although inadequate information is available on *CCNE1* (Cyclin E1) and Cyclin Dependent Kinase (*CDK*) gene in muscle tissues, both participate in the cell growth during the cell cycle^57,58^. *CDKL2* is known to be involved in cell proliferation in human cancer cells^59,60^, while *PER1* gene of the circadian clock is known to regulate cell proliferation and apoptosis in association with human cancer cells^61^. Further investigations are required to determine the precise role of these genes in sheep muscle development.

## Conclusion

This study is an attempt to gain an insight into the dynamics of gene expression with progression of age in sheep skeletal muscles. Our results highlight the temporal changes in gene expression in skeletal muscles from lambs to adult sheep. Noteworthy demarcation between lambs and adult sheep was observed in the expression of genes associated with muscle growth, adipogenesis and apoptosis. The enrichment of PI3K-Akt and MAPK pathways in lambs suggests their relevance in muscle growth. This differential expression analysis will contribute towards understanding the genetic basis of physiological changes in muscles with age. Several highly connected genes identified in our study are known to be involved in muscle development and proliferation and may serve as candidates for future research in myogenesis.

## Materials and methods

### Samples

Healthy animals were selected in coordination with the sheep rearers and butchers from Mandya district, Karnataka. As the animals were selected from the field, they were considered healthy if they did not show any visible signs of infection or ailment and were inspected by a veterinary officer. Care was taken to select unrelated male animals from flocks reared under the same management and feed. Since no records of the animals were maintained, the age of the animals was determined by information provided by the rearers as well as the dentine pattern. A two-tooth stage confirmed the age about 12 months (Group 1), 4-tooth between 18 and 24 months (Group 2), 6-tooth between 30 and 36 months (Group 3) and 8-tooth between 42 and 48 months (Group 4). Four animals in each group were selected. The animals were slaughtered according to standard commercial ‘*halal*’ procedures.

### RNA sequencing

The *longissimus thoracis* muscles were collected in RNA*Later* (Thermo Fisher Scientific Baltics, UAB, Vilnius, Lithuania) solution. RNeasy kit (Qiagen, Hilden, Germany) was used for extraction and purification of total RNA. Samples with RIN value ≥ 7.0 (Agilent Bioanalyzer) were selected for further processing. Libraries of four biological replicates from each age group were prepared by TruSeq RNA Library Prep Kit v2 (Illumina, San Diego, CA, USA). Paired end sequencing (150 bp) was performed on Illumina HiSeq-2000 Platform.

### Data analysis

FastQC (v 0.11.5) was used for assessment of the quality of samples^62^. The raw reads were trimmed and filtered using FastXToolKit. CLS Genomics Workbench 6.5.1 (CLC Bio, Aarhus, Denmark) was used for data analysis. The reads were mapped against the ovine genome assembly (Oar_v4.0). The RNA sequencing data have been deposited in the NCBI Bio Project PRJNA416678 with accession numbers SAMN16191735-750. Normalization of reads was done as reads per kilobase million (RPKM) and reads with RPKM values > 0.01 were included in the study. The CLC transcriptomics analysis tool was used for differential expression analysis across the different age groups.

### Validation by real time quantitative PCR (RT qPCR)

Ten differentially expressed genes were selected for RT qPCR analysis (Table S7). Primers for selected genes were designed by Primer 3 software^63^ or taken from published sequences^64–66^. cDNA was synthesized from 2 µg of purified RNA, using Super Script III Reverse Transcriptase (Thermo Fisher Scientific, Carlsbad, CA), as per manufacturer’s protocol. Each sample was analyzed in triplicate qPCR reactions. The final reaction volume of 10 µl contained 2 µl of cDNA, 8 µl of qPCR master mix (5 µl of SYBR Green Real-Time master mix (Applied Biosystems,Vilnius*, *Lithuania), 0.3 µl of each primer, 2.4 µl of DNA/RNA-free water). The samples were run on QuantStudio 5 Real-Time PCR System (Applied Biosystems). Standard curve calculation using four points of cDNA serial dilutions was used to estimate the PCR efficiency.

### Statistical analysis

Differentially expressed genes with log_2_ fold change ≥ 2.0 and p value (p_adj_) < 0.05 were used for further analyses. DAVID^67^, Consensus Pathway Data Base^68^ and Reactome were utilized for the functional and pathways analysis^69^. Cytoscapever 3.6.0^70^ along with cytoHubba app was utilized for gene-protein network analysis^71^. For the RT qPCR the mean cycle threshold (Ct) values of the genes were normalized to geometric mean of the reference genes *PPIB* and *β-ACTN*^72^. The 2^−ΔΔCT^ method was used for data analysis^73^.

### Ethics approval

Animal samples were purchased from local butchers. All ethical norms and guidelines were followed, with approval from Institutional Animal Ethics Committee, ICAR-National Bureau of Animal Genetic Resources, Karnal, Haryana, India (F.No. NBAGR/IAEC/2017, dated 21.01.2017).

## Supplementary Information

Supplementary Information.

## Data Availability

Data supporting this paper was generated by ICAR-NBAGR. The dataset generated in the study has been deposited in the NCBI (PRJNA416678).

## References

[1] BAHS-Basic Animal Husbandry & Fisheries Statistics, Government of India, Ministry of Agriculture, Department of Animal Husbandry, dairying & Fisheries, Krishi Bhavan, New Delhi, 1–132 (2019).

[2] Mullen AM, Stapleton PC, Corcoran D, Hamill RM, White A (2006). Understanding meat quality through the application of genomic and proteomic approaches. Meat. Sci..

[3] Kopuzlu S, Esenbuga N, Onenc A, Macit M, Yanar M, Yuksel S, Ozluturk A, Unlu N (2018). Effects of slaughter age and muscle type on meat quality characteristics of Eastern Anatolian Red bulls. Arch. Anim. Breed.

[4] Li Q, Wang Y, Tan L, Leng J, Lu Q, Tian S, Shao S, Duan C, Li W, Mao H (2018). Effects of age on slaughter performance and meat quality of Binlangjang male buffalo. Saudi J. Biol. Sci..

[5] Byrne K, Vuocolo T, Gondro C, White JD, Cockett NE, Hadfield T, Bidwell CA, Waddell JN, Tellam RL (2010). A gene network switch enhances the oxidative capacity of ovine skeletal muscle during late fetal development. BMC Genomics.

[6] Lin Y, Zhu J, Wang Y, Li Q, Lin S (2017). Identification of differentially expressed genes through RNA sequencing in goats (*Capra hircus*) at different postnatal stages. PLoS ONE.

[7] Sadkowski T, Jank M, Oprzadek J, Motyl T (2006). Age-dependent changes in bovine skeletal muscle transcriptomic profile. J. Physiol Pharmacol..

[8] Bongiorni S, Gruber CE, Bueno S, Chillemi G, Ferrè F, Failla S, Moioli B, Valentini A (2016). Transcriptomic investigation of meat tenderness in two Italian cattle breeds. Anim Genet..

[9] Ayuso M, Fernández A, Núñez Y, Benítez R, Isabel B, Barragán C, Fernández AI, Rey AI, Medrano JF, Cánovas A, González-Bulnes A, López-Bote C, Ovilo C (2015). Comparative analysis of muscle transcriptome between pig genotypes identifies genes and regulatory mechanisms associated to growth, fatness and metabolism. PLoS ONE.

[10] Kumar A, Kaur M, Ahlawat S, Sharma U, Singh MK, Singh KV, Chhabra P, Vijh RK, Yadav A, Arora R (2021). Transcriptomic diversity in *longissimus thoracis* muscles of Barbari and Changthangi goat breeds of India. Genomics.

[11] Hocquette JF, Bernard-Capel C, Vidal V, Jesson B, Levéziel H, Renand G, Cassar-Malek I (2012). The GENOTEND chip: A new tool to analyse gene expression in muscles of beef cattle for beef quality prediction. BMC Vet. Res..

[12] Arora R, Siddaraju NK, Sudarshan S, Fairoze MN, Kaur M, Sharma A, Girdhar Y, Sreesujatha RM, Devatkal SK, Ahlawat S, Vijh RK, Manjunatha SS (2019). Transcriptome profiling of *longissimus thoracis* muscles identifies highly connected differentially expressed genes in meat type sheep of India. PLoS ONE.

[13] Kaur M, Kumar A, Siddaraju NK, Fairoze MN, Chhabra P, Ahlawat S, Vijh RK, Yadav A, Arora R (2020). Differential expression of miRNAs in skeletal muscles of Indian sheep with diverse carcass and muscle traits. Sci. Rep..

[14] Zhao Y, Li J, Liu H, Xi Y, Xue M, Liu W, Zhuang Z, Lei M (2015). Dynamic transcriptome profiles of skeletal muscle tissue across 11 developmental stages for both Tongcheng and Yorkshire pigs. BMC Genomics.

[15] Weston AR, Rogers Pas RW, Althen TG (2002). The role of collagen in meat tenderness. Profess. Anim. Sci..

[16] Polidori P, Pucciarelli S, Cammertoni N, Polzonetti V, Vincenzetti S (2017). The effects of slaughter age on carcass and meat quality of Fabrianese lambs. Small Rumin. Res..

[17] Hogarth MW, Garton FC, Houweling PJ, Tukiainen T, Lek M, Macarthur DG, Seto JT, Quinlan KG, Yang N, Head SI, North KN (2016). Analysis of the ACTN3 heterozygous genotype suggests that α-actinin-3 controls sarcomeric composition and muscle function in a dose-dependent fashion. Hum. Mol. Genet..

[18] Lee LA, Karabina A, Broadwell LJ, Leinwand LA (2019). The ancient sarcomeric myosins found in specialized muscles. Skelet. Muscle..

[19] Gomes AV, Potter JD, Szczesna-Cordary D (2002). The role of troponins in muscle contraction. IUBMB Life.

[20] Johnston JR, Chase PB, Pinto JR (2018). Troponin through the looking-glass: Emerging roles beyond regulation of striated muscle contraction. Oncotarget.

[21] Wojtysiak D, Kaczor U, Połtowicz K, Krzysztoforski K (2010). The effects of sex and slaughter weight on muscle fibre characteristics and physico-chemical properties of lamb *longissimus thoracis* muscle. Anim. Sci. Papers Rep..

[22] Lian T, Wang L, Liu Y (2013). A new insight into the role of calpains in post-mortem meat tenderization in domestic animals: A review. Asian-Australas. J. Anim. Sci..

[23] Cruzen SM, Paulino PV, Lonergan SM, Huff-Lonergan E (2014). Postmortem proteolysis in three muscles from growing and mature beef cattle. Meat Sci..

[24] Nixon RA (2003). The calpains in aging and aging-related diseases. Ageing Res Rev..

[25] Dimitrova Y, Gruber AJ, Mittal N, Ghosh S, Dimitriades B, Mathow D, Grandy WA, Christofori G, Zavolan M (2017). TFAP2A is a component of the ZEB1/2 network that regulates TGFB1-induced epithelial to mesenchymal transition. Biol. Direct..

[26] Muñoz-Cánoves P, Scheele C, Pedersen BK, Serrano AL (2013). Interleukin-6 myokine signaling in skeletal muscle: A double-edged sword?. FEBS J..

[27] Baeza-Raja B, Munoz-Canoves P (2004). p38 MAPK-induced nuclear factor-kappa B activity is required for skeletal muscle differentiation: Role of interleukin-6. Mol. Biol Cell..

[28] Mukund K, Subramaniam S (2020). Skeletal muscle: A review of molecular structure and function, in health and disease. Wiley Interdiscip. Rev. Syst. Biol. Med..

[29] Cassar-Malek I, Passelaigue F, Bernard C, Léger J, Hocquette JF (2007). Target genes of myostatin loss-of-function in muscles of late bovine fetuses. BMC Genomics.

[30] Goldspink G, Fernandes K, Williams PE, Wells DJ (1994). Age-related changes in collagen gene expression in the muscles of mdx dystrophic and normal mice. Neuromuscul. Disord..

[31] McBryan J, Hamill RM, Davey G, Lawlor P, Mullen AM (2010). Identification of suitable reference genes for gene expression analysis of pork meat quality and analysis of candidate genes associated with the trait drip loss. Meat Sci..

[32] Hamill RM, Aslan O, Mullen AM, O'Doherty JV, McBryan J, Morris DG, Sweeney T (2013). Transcriptome analysis of porcine M semimembranosus divergent in intramuscular fat as a consequence of dietary protein restriction. BMC Genomics.

[33] Mudry JM, Massart J, Szekeres FL, Krook A (2015). TWIST1 and TWIST2 regulate glycogen storage and inflammatory genes in skeletal muscle. J. Endocrinol..

[34] Miraoui H, Marie PJ (2010). Pivotal role of twist in skeletal biology and pathology. Gene.

[35] Cortez-Toledo O, Schnair C, Sangngern P, Metzger D, Chao LC (2017). Nur77 deletion impairs muscle growth during developmental myogenesis and muscle regeneration in mice. PLoS ONE.

[36] Pan X, Liu B, Chen S, Ding H, Yao X, Cheng Y, Xu D, Yin Y, Dai X, Sun J, Xu G, Pan J, Xiao L, Xie L (2019). Nr4a1 as a myogenic factor is upregulated in satellite cells/myoblast under proliferation and differentiation state. Biochem. Biophys. Res. Commun..

[37] Jean-Baptiste G, Yang Z, Khoury C, Gaudio S, Greenwood MT (2005). Peptide and non-peptide G-protein coupled receptors (GPCRs) in skeletal muscle. Peptides.

[38] Collins BC, Mader TL, Cabelka CA, Iñigo MR, Spangenburg EE, Lowe DA (2018). Deletion of estrogen receptor α in skeletal muscle results in impaired contractility in female mice. J. Appl. Physiol..

[39] Lee SJ, Lee EJ, Kim SH, Choi I, Lee DM, Lee HJ, Yoon D, Chun T (2011). IL-17A promotes trans differentiation of mouse myoblast cells (C2C12) into adipocytes by increasing the expression of peroxisome proliferator-activated receptor γ through CAAT/enhancer binding protein β signaling. Biotechnol. Lett..

[40] Khan R, Raza SHA, Guo H, Xiaoyu W, Sen W, Suhail SM, Rahman A, Ullah I, Abd El-Aziz AH, Manzari Z, Alshawi A, Zan L (2020). Genetic variants in the TORC2 gene promoter and their association with body measurement and carcass quality traits in Qinchuan cattle. PLoS ONE.

[41] Umansky KB, Gruenbaum-Cohen Y, Tsoory M, Feldmesser E, Goldenberg D, Brenner O, Groner Y (2015). Runx1 transcription factor is required for myoblasts proliferation during muscle regeneration. PLoS Genet..

[42] Kuang S, Kuroda K, Le Grand F, Rudnicki MA (2007). Asymmetric self-renewal and commitment of satellite stem cells in muscle. Cell.

[43] Ganassi M, Badodi S, Wanders K, Zammit PS, Hughes SM (2020). Myogenin is an essential regulator of adult myofibre growth and muscle stem cell homeostasis. Elife.

[44] Choi IY, Lim H, Cho HJ, Oh Y, Chou BK, Bai H, Cheng L, Kim YJ, Hyun S, Kim H, Shin JH, Lee G (2020). Transcriptional landscape of myogenesis from human pluripotent stem cells reveals a key role of TWIST1 in maintenance of skeletal muscle progenitors. Elife.

[45] Kokabu S, Nakatomi C, Matsubara T, Ono Y, Addison WN, Lowery JW, Urata M, Hudnall AM, Hitomi S, Nakatomi M, Sato T, Osawa K, Yoda T, Rosen V, Jimi E (2017). The transcriptional co-repressor TLE3 regulates myogenic differentiation by repressing the activity of the MyoD transcription factor. J. Biol. Chem..

[46] Swiderski K, Caldow MK, Naim T, Trieu J, Chee A, Koopman R, Lynch GS (2019). Deletion of suppressor of cytokine signaling 3 (SOCS3) in muscle stem cells does not alter muscle regeneration in mice after injury. PLoS ONE.

[47] Nikawa T, Ishidoh K (2020). Ubiquitin ligase Cbl-b and inhibitory Cblin peptides. Biochim. Biophys. Acta Proteins Proteom..

[48] Tower J (2009). Hsps and aging. Trends Endocrinol. Metab..

[49] Mymrikov EV, Seit-Nebi AS, Gusev NB (2011). Large potentials of small heat shock proteins. Physiol. Rev..

[50] Malheiros JM, Enríquez-Valencia CE, da Silva Duran BO, de Paula TG, Curi RA, de Vasconcelos Silva JAII, Dal-Pai-Silva M, de Oliveira HN, Chardulo LAL (2018). Association of CAST2, HSP90AA1, DNAJA1 and HSPB1 genes with meat tenderness in Nellore cattle. Meat Sci..

[51] Picard B, Berri C, Lefaucheur L, Molette C, Sayd T, Terlouw CC (2010). Skeletal muscle proteomics in livestock production. Brief Funct. Genomics.

[52] Guillemin N, Jurie C, Cassar-Malek I, Hocquette JF, Renand G, Picard B (2011). Variations in the abundance of 24 protein biomarkers of beef tenderness according to muscle and animal type. Animal.

[53] Ueda S, Kokaji Y, Simizu S, Honda K, Yoshino K, Kamisoyama H, Shirai Y, Yamanoue M (2015). Chicken heat shock protein HSPB1 increases and interacts with αB-crystallin in aged skeletal muscle. Biosci. Biotechnol. Biochem..

[54] Carra S, Seguin SJ, Landry J (2008). HspB8 and Bag3: A new chaperone complex targeting misfolded proteins to macro autophagy. Autophagy.

[55] Miguel E, Blázquez B, Ruiz de Huidobro F (2021). Live weight and sex effects on instrumental meat quality of Rubia de El Molar autochthonous ovine breed. Animals.

[56] Walsh RJ, Kong SW, Yao Y, Jallal B, Kiener PA, Pinkus JL, Beggs AH, Amato AA, Greenberg SA (2007). Type I interferon-inducible gene expression in blood is present and reflects disease activity in dermato myositis and poly myositis. Arthritis Rheum..

[57] Morgan DO (1995). Principles of CDK regulation. Nature.

[58] Winey M (1999). Cell cycle: Driving the centrosome cycle. Curr Biol..

[59] Li L, Liu C, Amato RJ, Chang JT, Du G, Li W (2014). CDKL2 promotes epithelial-mesenchymal transition and breast cancer progression. Oncotarget.

[60] Fang CL, Uen YH, Chen HK, Hseu YC, Lin CC, Hung ST, Sun DP, Lin KY (2018). Loss of cyclin-dependent kinase-like 2 predicts poor prognosis in gastric cancer, and its overexpression suppresses cells growth and invasion. Cancer Med..

[61] Gong X, Tang H, Yang K (2021). PER1 suppresses glycolysis and cell proliferation in oral squamous cell carcinoma via the PER1/RACK1/PI3K signaling complex. Cell Death Dis..

[62] Andrews, S. FastQC: A quality control tool for high throughput sequence data. http://www.bioinformatics.babraham.ac.uk/projects/fastqc (2010).

[63] Untergasser A, Cutcutache I, Koressaar T, Ye J, Faircloth BC, Remm M, Rozen SG (2012). Primer3-new capabilities and interfaces. Nucleic Acids Res..

[64] Banerjee D, Upadhyay RC, Chaudhary UB, Kumar R, Singh S, Ashutosh GJM, Polley S, Mukherjee A, Das TK, De S (2014). Seasonal variation in expression pattern of genes under *HSP70* family in heat- and cold-adapted goats (*Capra hircus)*. Cell Stress Chaperones.

[65] Bernard C, Cassar-Malek I, Le Cunff M, Dubroeucq H, Renand G, Hocquette JF (2007). New indicators of beef sensory quality revealed by expression of specific genes. J. Agric. Food Chem..

[66] Zhu W, Lin Y, Liao H, Wang Y (2015). Selection of reference genes for gene expression studies related to intramuscular fat deposition in *Capra hircus* skeletal muscle. PLoS ONE.

[67] Huang DW, Sherman BT, Lempicki RA (2009). Systematic and integrative analysis of large gene lists using DAVID bioinformatics resources. Nat. Protoc..

[68] Kamburov A, Pentchev K, Galicka H, Wierling C, Lehrach H, Herwig R (2011). ConsensusPathDB: Toward a more complete picture of cell biology. Nucleic Acids Res..

[69] Jassal B, Matthews L, Viteri G, Gong C, Lorente P, Fabregat A, Sidiropoulos K, Cook J, Gillespie M, Haw R, Loney F, May B, Milacic M, Rothfels K, Sevilla C, Shamovsky V, Shorser S, Varusai T, Weiser J, Wu G, Stein L, Hermjakob H, D'Eustachio P (2020). The reactome pathway knowledge base. Nucleic Acids Res..

[70] Shannon P, Markiel A, Ozier O, Baliga NS, Wang JT, Ramage D, Amin N, Schwikowski B, Ideker T (2003). Cytoscape: A software environment for integrated models of biomolecular interaction networks. Genome Res..

[71] Chin CH, Chen SH, Wu HH, Ho CW, Ko MT, Lin C (2014). cytoHubba: Identifying hub objects and sub-networks from complex interactome. BMC Syst. Biol..

[72] Vandesompele J, De Preter K, Pattyn F, Poppe B, Van Roy N, De Paepe A, Speleman F (2002). Accurate normalization of real-time quantitative RT-PCR data by geometric averaging of multiple internal control genes. Genome Biol..

[73] Livak KJ, Schmittgen TD (2001). Analysis of relative gene expression data using real-time quantitative PCR and the 2(-delta deltaC(T)) method. Methods.

